# Endophytic fungal isolate mediated biosynthesis of silver nanoparticles and their free radical scavenging activity and anti microbial studies

**DOI:** 10.1007/s13205-016-0433-7

**Published:** 2016-06-10

**Authors:** Vasudeva Reddy Netala, Venkata Subbaiah Kotakadi, Pushpalatha Bobbu, Susmila Aparna Gaddam, Vijaya Tartte

**Affiliations:** 1 Department of Biotechnology, Sri Venkateswara University, Tirupati, AP India; 2 DST-PURSE Centre, Sri Venkateswara University, Tirupati, 517502 AP India; 3 Department of Virology, Sri Venkateswara University, Tirupati, AP India; 4 Department of Botany, Sri Venkateswara University, Tirupati, 517502 AP India

**Keywords:** 18S rRNA gene, *Aspergillus versicolor*, AgNPs, Zeta potential, Free radical scavenging activity, Antimicrobial activity

## Abstract

The present study reports that the biosynthesis of AgNPs using an endophytic fungus isolated from the ethnomedicinal plant *Centella asiatica*. The endophytic fungus was identified as *Aspergillus versicolor* ENT7 based on 18S rRNA gene sequencing (NCBI Accession number KF493864). The AgNPs synthesized were characterized by UV–visible spectroscopy, Fourier transform infra-red spectroscopy (FTIR), transmission electron microscopy (TEM), X-ray diffraction (XRD), particle size analyzer, and zeta potential measurements. The UV–Vis absorption spectra showed the peak at 429 nm which confirmed the synthesis of AgNPs. TEM analysis revealed that the AgNPs were spherical in shape with 3–40 nm in size; similar results were also obtained by Horiba particle size analyzer with 5–40 nm in size. The synthesized AgNPs were highly stable due to their high negative zeta potential value of −38.2 mV. XRD studies showed (111), (200), (220), (311), and (222) planes of the face-centered cubic (FCC) lattice, indicating the crystalline nature of the AgNPs. Selected area electron diffraction (SAED) pattern of the AgNPs showed five circular fringes which were in accordance with XRD data and confirmed the formation of high crystalline nature of AgNPs. FTIR measurements indicated the peaks at 3273, 2925, 1629, 1320, and 1020 cm^−1^ corresponding to different functional groups possibly involved in the synthesis and stabilization of AgNPs. The synthesized AgNPs exhibited effective free radical scavenging activity with the IC50 value of 60.64 µg/ml. The synthesized AgNPs were found to be highly toxic against both gram-positive and gram-negative bacteria and also showed a very good antifungal activity.

## Introduction

Over the last decade majority of the researchers shifted their focus to nanometer-sized particles, particularly metal nanoparticles because of their controllable size and shape, ease of synthesis, and strong optical properties. The unique size- and shape-dependent properties of metal nanoparticles have significantly impacted all spheres of human life and making nanobiotechnology a promising field (Ravindran et al. 2013). Nanobiotechnology find innumerable applications due to strong optical absorbance and surface bioconjugation with molecular biomarkers related to surface plasmon resonance effect (Kreibig and Vollmer 1995). Noble metal nanoparticles particularly silver nanoparticles (AgNPs) have gained a significant interest in the recent years, owing to their remarkable optical, optoelectronic, magnetic, catalytic, and thermal properties and thus find applications as optical receptors (Karimzadeh and Mansour 2010), sensors (Cobley et al. 2009), catalysts in chemical reactions (Edison and Sethuraman 2012), signal enhancers in SERS-based enzyme Immunoassay (Chen et al. 2009), DNA detection and delivery, bio-analyzers (Ravindran et al. 2013), Cytotoxic (Asad et al. 2013), free radical scavenging (Ramamurthy et al. 2013), and antimicrobial agents (Netala et al. 2014, 2015a; Kotakadi et al. 2013).

Several physical and chemical approaches have been reported for the biosynthesis of silver nanoparticles, including thermal decomposition (Plante et al. 2010), laser ablations (Simakin et al. 2004), gamma radiation assisted (Chol et al. 2005), polyol assisted, and chemical reduction methods (Frattine et al. 2005). Irradiation and other physical methods are not environmental friendly and also possess health risks. Hazardous chemicals, such as hydroxylamine, poly-*N*-vinyl pyrrolidine, polyvinyl alcohol, and sodium borohydride, are involved in the chemical approaches. The application of chemical approaches is not eco-friendly and possesses biological risks, and the toxic chemicals on the surface limit the applications of AgNPs in pharmaceutical and biomedical fields. Hence, the biological methods for the synthesis of AgNPs have gained a significant interest in the field of nanotechnology. Supramolecular complexes of biomolecules and their topographic and electrostatic properties are being widely employed in biomedical and pharmaceutical fields. Peptides to proteins, sugars to polysaccharides, isoprenes to terpenoids, polyphenols, glycosides, plant and microbial derived compounds, viral particles, etc., are being constantly explored for the biosynthesis of metal nanoparticles and novel carriers. Biological approaches are simple, rapid, and cost effective and involve the synthesis of non-toxic, clean, and biocompatible AgNPs. Fungal mediated synthesis of AgNPs has recently emerged as a new avenue in the field of nanobiotechnology. Compared to bacteria, fungi are more advantageous, because they grow at fast rates and very easy to culture, and maintain in the laboratory. Fungi being eukaryotes produce high amounts of proteins and other biomolecules. These proteins and biomolecules will often associate with nanoparticles that must be preventing the agglomeration and stabilize nanoparticles. Various fungi have been reported to synthesize AgNPs, including *Alternaria alternate* (Monali et al. 2009), *Amylomyces rouxii* (Javed et al. 2010), *Aspergillus niger* (Netala et al. 2015b), *Coriolus versicolor* (Rashmi and Varma 2009), *Fusarium solani* (Rafie et al. 2012), *Humicola sp* (Asad et al. 2013), *Neurospora crossa* (Longoria et al. 2011), *Schizophyllum radiatum* (Metuku et al. 2014), and *Trichoderma viride* (Fayaz et al. 2009) have been reported.

Endophytic fungi are the intriguing group of fungal species which colonise living and healthy tissues of plants. Endophytic fungi produce natural bioactive compounds which are considered to be alternative sources for plant producing bioactive compounds (Strobel et al. 2002). Exploration of endophytic fungi for the biosynthesis of AgNPs is considered as their another important application in the pharmaceutical and biomedical field. In the present study, we report the biosynthesis of AgNPs using extracellular filtrate of endophytic fungus *Aspergillus versicolor* ENT7 strain isolated from the healthy leaf tissues of *Centella asiatica,* an important medicinal plant which harbors many endophytic fungi. The AgNPs were characterized using different measurements which include UV–Vis, FTIR, XRD, TEM, particle size analyzer, and zeta potential measurements. Biomedical importance of the AgNPs was evaluated by checking for free radical scavenging and antimicrobial activities.

## Materials and methods

### Isolation of endophytic fungi

Mature leaves of *Centella asiatica* were collected from plants grown in the Sri Venkateswara University campus, Tirupati, A.P. India. Leaves were washed under running tap water and then with teepol solution to ensure for dust free and clean. Then, leaves were washed thoroughly with sterile double distilled water (SDDW). Under aseptic conditions, leaves were surface sterilized with 10 % H2O2 and then with 80 % alcohol followed by thorough rinsing with SDDW for three to four times. Leaves were dried on sterile blotting paper and then cut into small segments. Leaf segments were placed on solidified potato dextrose agar (PDA) plates. PDA plates were incubated at 24 ± 2 °C for 10 days. After 10 days, fungal mycelia were harvested and transferred onto fresh PDA plates. Pure fungal cultures were then identified by 18S rRNA gene amplification and sequencing.

### Identification of endophytic fungi

#### DNA extraction

For the extraction of genomic DNA, 50 mg of fungal mycelia was frozen in liquid nitrogen and mechanically disrupted. The extraction of genomic DNA was carried out using Qiagen kit (USA) according to manufacturer’s instructions.

#### PCR amplification and sequencing of 18S rRNA gene

The ITS region (ITS 1-5.8S-ITS 2) of 18S rRNA gene was amplified using fungal domain specific primers ITS1–5′-TCCGTAGGTGAACCTGCGG-3′ (forward primer) and ITS4–5′-TCCTCCGCTTATTGATATGC-3′(reverse primer). Amplification was performed in a 50 ul reaction mixture containing 50 ng of template DNA, 200 μM each dNTP, 1.5 mM MgCl_2_, 20 pmol of each primer, and 0.4 U of Taq DNA polymerase in a CG palm cycler (Genetix biotech asia). The amplification cycles consisted of the initial denaturation at 94 °C for 5 min followed by 30 cycles of 94 °C for 45 s, 55 °C for 1 min, 72 °C for 1 min, and a final extension at 72 °C for 7 min. The amplification was confirmed by running the amplified product in 1.2 % w/v agarose gel electrophoresis with ethidium bromide staining and documented by gel documentation system (Major science, UVDI). The PCR products were purified using gel extraction kit. Sequencing was carried out at MWGAG Biotech, Bangalore, India. The sequence obtained was analyzed by BLASTn and NCBI and was identified by homology search for closely related sequence. Multiple sequence alignment was carried out using ClustalW2, and phylogenetic tree was constructed using the neighbor-joining (NJ) method.

#### Biosynthesis of AgNPs

The synthesis of silver nanoparticles was carried out according to the method described earlier (Jaidev and Narasimha 2010). The fungal isolate *Aspergillus versicolor* was cultured in 100 ml of potato dextrose broth and was incubated at 26 ± 2 °C in shaking incubator (LABLINE) at a speed of 100 rpm. After 7 days of growth, the fungal biomass was harvested and washed thoroughly with SDDW to prevent the contamination of medium components. 10 g of fungal biomass was taken in 250 ml Erlenmeyer flask containing 100 ml SDDW and incubated at 28 ± 4 °C for 72 h in shaking incubator at the speed of 100 rpm. After incubation, the aqueous solution was separated by filtration through Whatmann No.1 filter paper. This solution, namely, fungal filtrate, used for the synthesis of AgNPs. 100 ml of 1 mM of silver nitrate was then added to 100 ml of fungal filtrate and incubated at 28 ± 4 °C for 24 h in the dark condition.

#### Characterization of AgNPs

The synthesis of AgNPs was confirmed by the absorbance spectrum using UV–Vis spectrometer (Analytical Technologies Ltd, India). The spectrum was recorded with a resolution of 1 nm between 200 and 700 nm. The synthesized AgNPs were purified by the centrifugation of solution of AgNPs at 15,000 rpm for 15 min thrice with continuous washing the pellet with sterile Milli Q water. The final pellet that obtained was dried in hot air oven at 50 °C for 24 h, and the pure powder obtained was used for FTIR, XRD, TEM, and other studies. FTIR analysis was carried out to study the functional groups possibly involved in the synthesis and stabilization of AgNPs. FTIR spectrum (Model ALPHA interferometer) was recorded in the range of 500–4000 cm^−1^ with the resolution of 2 cm^−1^. XRD patterns were obtained on an Ultima IV X-ray powder diffractometer (Rigaku, Tokyo, Japan) using CuKα radiation (*λ* = 1.5406). The shape and size of the AgNPs were determined using TEM (FEI Tecnai F12, Philips Electron Optics, Holland) operated at 100 kV. Particle size and zeta potential experiments were carried out using a Horiba Nanopartica instrument.

#### Free radical scavenging activity

The free radical scavenging activity of the AgNPs was measured in vitro by 2, 2′-diphenyl-1-picrylhydrazyl (DPPH) radical scavenging assay according to the method described earlier (Mittal et al. 2006). The stock solution was prepared by dissolving 4 mg of DPPH in 100 ml of methanol and stored at 20 °C. 2 ml of this solution was added to 1 ml of methanol solution containing tested samples of different concentrations (25, 50, 75, and 100 μg/ml). Ascorbic acid was used as standard. DPPH radical scavenging activity (RSA) was measured by the absorbance at 517 nm

$${\text{RSA }}\left( \% \right) = [({\text{control absorbance}}{-}{\text{sample absorbance}})/\left( {\text{control absorbance}} \right)] \times 100.$$

#### Antimicrobial activity

Antimicrobial activity of the biosynthesized AgNPs was checked against both bacterial and fungal pathogens. Antibacterial activity of the AgNPs was checked against most pathogenic *Staphylococcus aureus, Streptococcus pneumonia* (gram positive), *Pseudomonas aeruginosa*, and *Klebsiella pneumonia* (gram negative) by employing the disc diffusion method using 24 h active cultures (Pulicherla et al. 2015). In the present study, bacterial cultures showed that the optical density (OD) value of 0.2 at 600 nm wavelength, according to McFarland calculation bacterial cell density, in this study is determined as 6 × 10^7^ CFU/ml. 200 µl of bacterial inoculum was spread on nutrient agar (NA) plates. Sterile paper disc dipped in 25 µl (1 mg/1 ml) of AgNPs solution was placed on agar plates. Streptomycin was used as the standard antibiotic. NA plates were incubated at 37 °C for 24 h and observed for the zone of inhibition (ZOI).

Antifungal activity of the AgNPs was tested against the fungus *Candida albicans* and *Candida nonalbicans* using the disc diffusion method (Pulicherla et al. 2015). 200 µl of fungal inoculum was spread on the potato dextrose agar (PDA) plates. Sterile paper disc impregnated with 25 µL of AgNPs solution (1 mg/1 ml) was placed on the PDA plate. Voriconazole was used as positive control. PDA plates were incubated at 25 °C for 72 h to observe the inhibition zones.

## Results and discussion

### Fungal identification using ITS rRNA gene sequencing and phylogenetic analysis

**Fig. 1:**
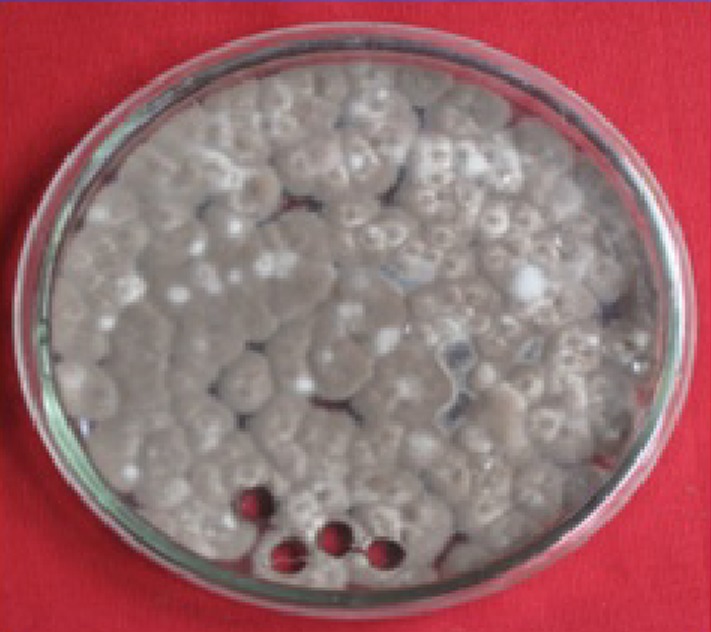
Colony morphology of *Aspergillus versicolor* strain ENT 7 isolated from the leaves of *Centella asiatica*

**Fig. 2:**
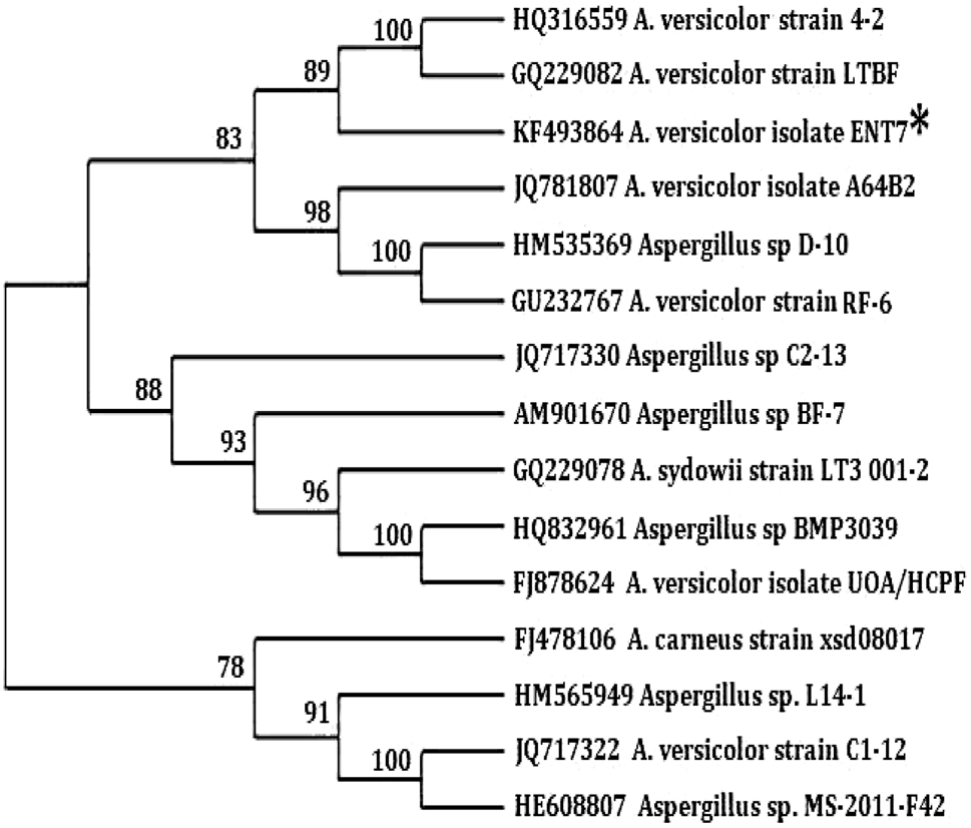
Phylogenetic analysis of 18S rRNA gene of *Aspergillus versicolor* strain ENT 7 with other fungal isolates. [neighbour-joining (NJ) analyses of phylogenetic relationship between the *A. versicolor* isolate ENT7 (KF493864) and ITS-rDNA of related fungal strains retrieved from NCBI GenBank]

The isolated endophytic fungus appeared as whitish brown in color (Fig. 1) and was characterized by the PCR amplification of 18S rRNA gene using ITS primers. The amplified PCR product was around the size of 500bps. The Sanger’s dideoxy nucleotide sequencing of amplified ITS region (ITS 1-5.8S-ITS 2) of 18 s rRNA gene resulted in 517 bp nucleotide sequence. The Blastn analyses, pairwise and multiple sequence alignment revealed 98–100 % identity with the sequences of *A. versicolor* strains and is designated as *Aspergillus versicolor* ENT 7 and has been deposited in NCBI GenBank (Accession Number KF493864). Multiple sequence alignment was carried out using ClustalW2 with default parameters. Phylogenetic tree was constructed by the neighbour-joining (NJ) method with nucleotide pairwise genetic distance corrections. Bootstrap test of phylogeny was carried out to check the reliability of tree topology as a percentage of 1000 replications. All branches with <70 % bootstrap support were collapsed (Fig. 2).

### Biosynthesis of AgNPs

**Fig. 3:**
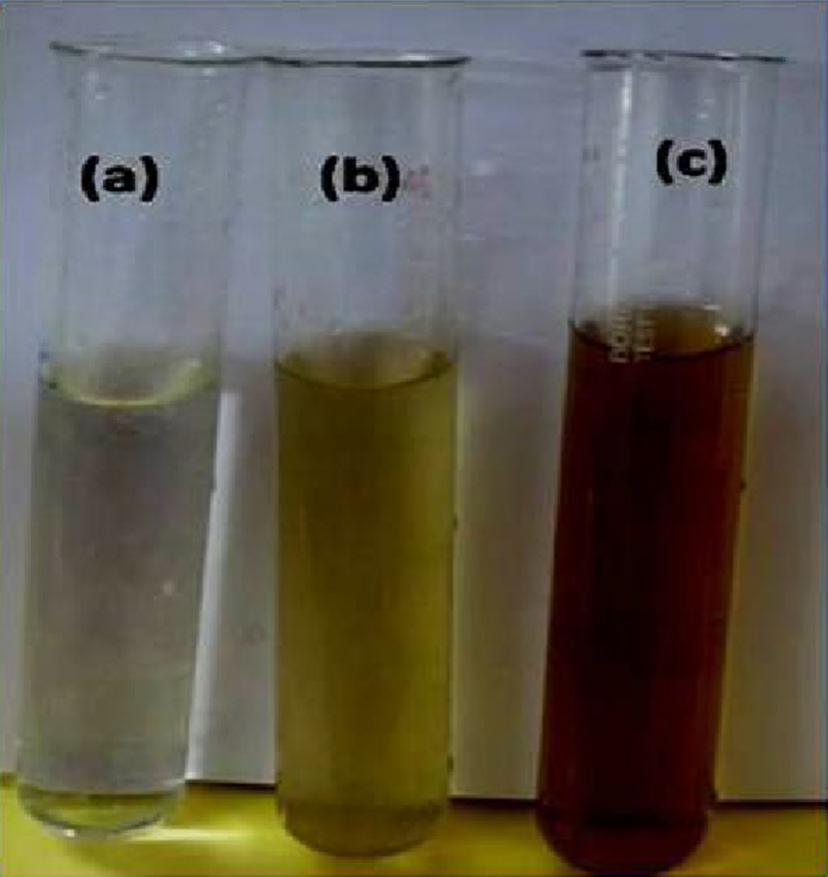
**a** 1 mM of AgNO_3_ solution. **b** *Light yellow color* of the reaction solution containing 1 mM AgNO_3_ and fungal filtrate. **c** Color change of the reaction solution from *light yellow* to *dark brown* indicating the formation of AgNPs

The synthesis of AgNPs by fungal filtrate of *A. versicolor* ENT 7 was investigated. After 24 h of incubation, light yellow color of the reaction mixture turned to dark brown color (Fig. 3). The color change indicated the reduction of silver ions (Ag^+^) into AgNPs (Ag^0^). It is well known that AgNPs exhibit a dark brown color in aqueous solution due to surface plasmon resonance (SPR). The fascinating and bright colors of metal nanoparticles are related to the localized surface plasmon resonance (SPR). The applications of metal nanoparticles have broadened due to surface bioconjugation with molecular biomarkers strong optical absorbance related to SPR (Kreibig and Vollmer 1995; Kelly et al. 2002).

### UV–visible spectra analysis

**Fig. 4:**
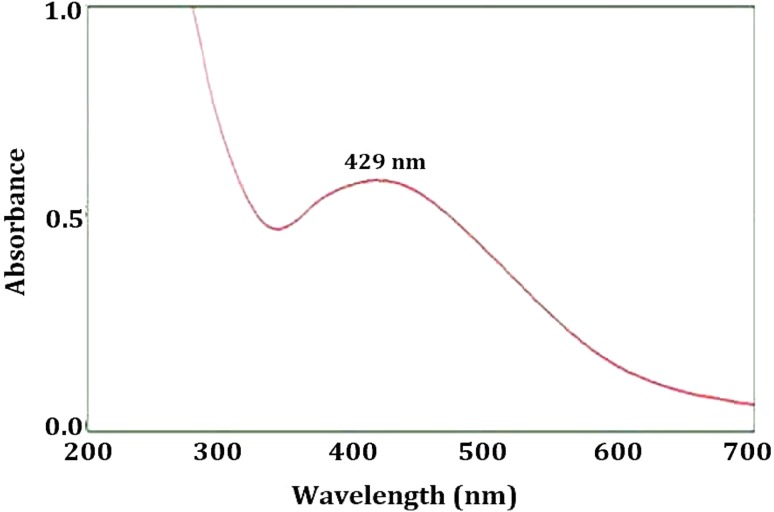
UV–Vis analysis of the synthesized AgNPs showing SPR peak at 429 nm

The UV–visible spectra of synthesized AgNPs (Fig. 4) **s**howed characteristic SPR peak at 429 nm. SPR peak at 429 nm confirmed the synthesis of AgNPs and is also responsible for the spherical shape of AgNPs synthesized. The shape of the synthesized AgNPs is further confirmed by the TEM analysis. Different metabolites and proteins present in the extracellular fungal filtrate could be responsible for the synthesis and stabilization of AgNPs, which is further confirmed by the FTIR analysis.

### FTIR spectra analysis

**Fig. 5:**
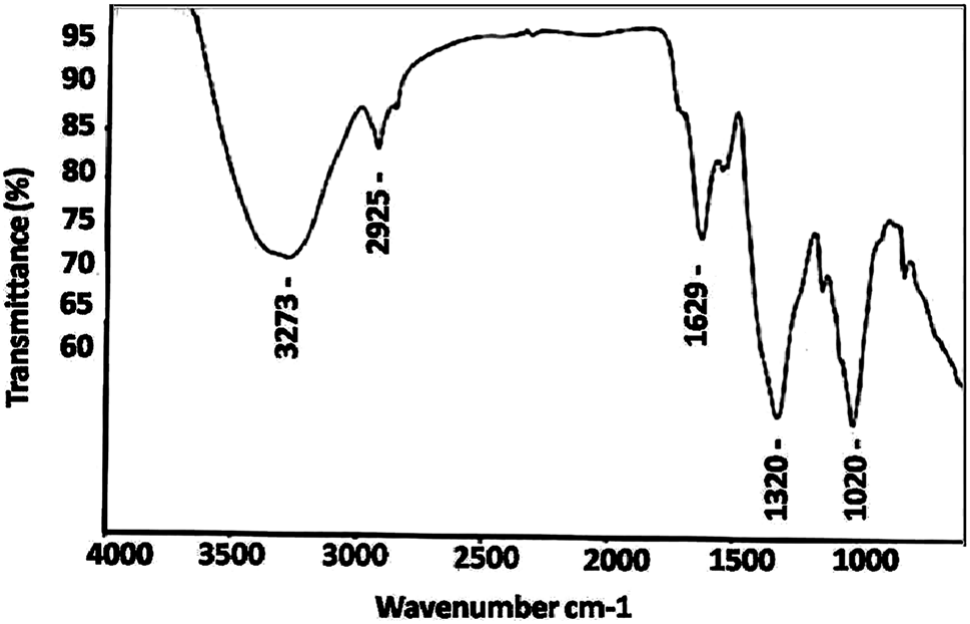
FTIR analysis of biosynthesized AgNPs

The FTIR analysis (Fig. 5) showed intensive peaks at 3273, 2925, 1629, 1320, and 1020 cm^−1^. The peak at 3273 cm^−1^ corresponding to N–H stretching of the secondary amide of the protein and the peak at 2925 cm^−1^ corresponding to C–H stretching of methylene groups of the protein (Bozanic et al. 2010). Peak at 1629 cm^−1^ corresponding to –CO stretch of amide I band of proteins (Fayaz et al. 2009). The sharp peak at 1320 cm^−1^ can be assigned to C–N stretching vibrations of aromatic amines (Rashmi and Varma 2009). The extracellular proteins present in the fungal filtrate could be responsible for the reduction of silver ions, Ag^+^ into nanosize silver particles, Ag^0^. These proteins have strong ability to bind silver nanoparticles, acts as capping agents and thus provided the stability to them. These results are consisted with the earlier reports for the fungal mediated synthesis of AgNPs (Jaidev and Narasimha 2010; Rafie et al. 2012: Asad et al. 2013). The peak at 1020 cm^−1^ can be assigned to C–OH of the phenols, supporting the participation of polyphenols, such as flavanoids and triterpenoids, in the reduction of Ag^+^ into Ag^0^ (Litvin et al. 2012; Litvin and Minaev 2013). FTIR analysis revealed that the polyphenols could act as bioreducing agents, while proteins could play a dual role as bioreducing and stabilizing agents.

### XRD analysis

**Fig. 6:**
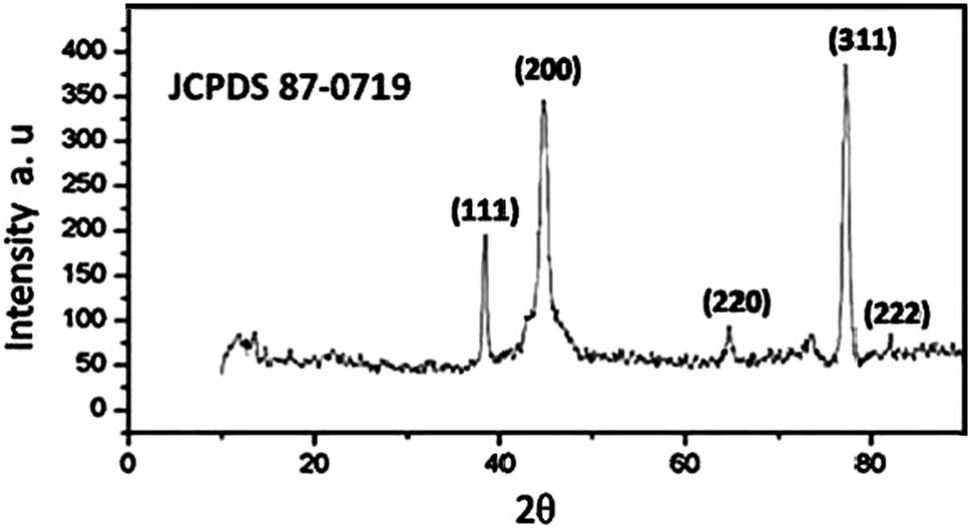
XRD pattern of biosynthesized AgNPs

The XRD pattern of the biosynthesized AgNPs was shown in Fig. 6. XRD pattern revealed four diffraction peaks at 38.31°, 44.58°, 64.71°, 77.71°, and 81.92° could be indexed to (111), (200), (220), (311), and (222) planes, respectively. All the peaks corresponding to face-centered cubic (FCC) lattice phase of silver and were consisted with the standard JCPDS (File No 87-0719) data. Thus, XRD pattern obtained for the AgNPs that revealed the crystalline nature of AgNPs with FCC phase and consisted with many earlier reports of the AgNPs synthesized by fungal extracts (Rafie et al. 2012; Asad et al. 2013).

### TEM analysis

**Fig. 7:**
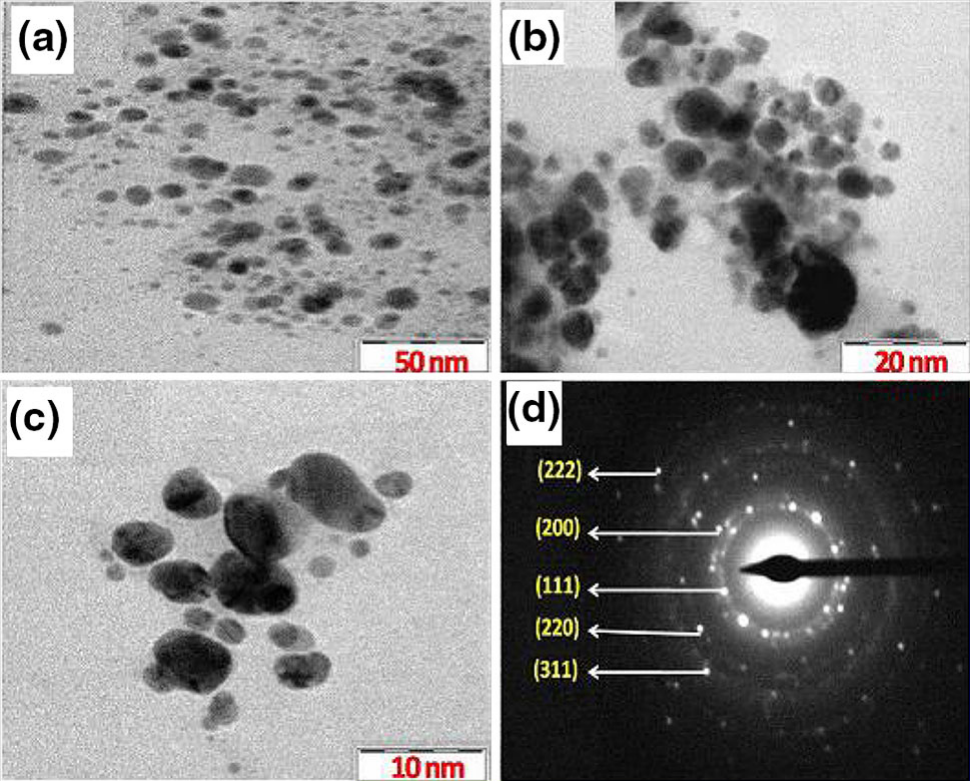
TEM images of AgNPs at **a** 50 nm **b** 20 nm and **c** 10 nm and corresponding SAED pattern **d** showed five diffraction rings

TEM analysis was performed to determine the size and shape of the biosynthesized AgNPs. TEM micrographs were represented at different resolutions (Fig. 7a, c). TEM micrographs obtained shows that AgNPs were roughly spherical in shape and well dispersed with the sizes ranging from 3 to 40 nm. From the TEM micrographs, it is evident that the size and shape of the AgNPs were fairly uniform. Selected area electron diffraction (SAED) pattern of the AgNPs (Fig. 7d) depicted circular fringes corresponding to (111), (200), (220), (311), and (222) planes which were in accordance with XRD data and further confirmed the formation of high crystalline AgNPs. The results of the TEM studies are in line with earlier reports (Metuku et al. 2014).

### Particle size determination

**Fig. 8:**
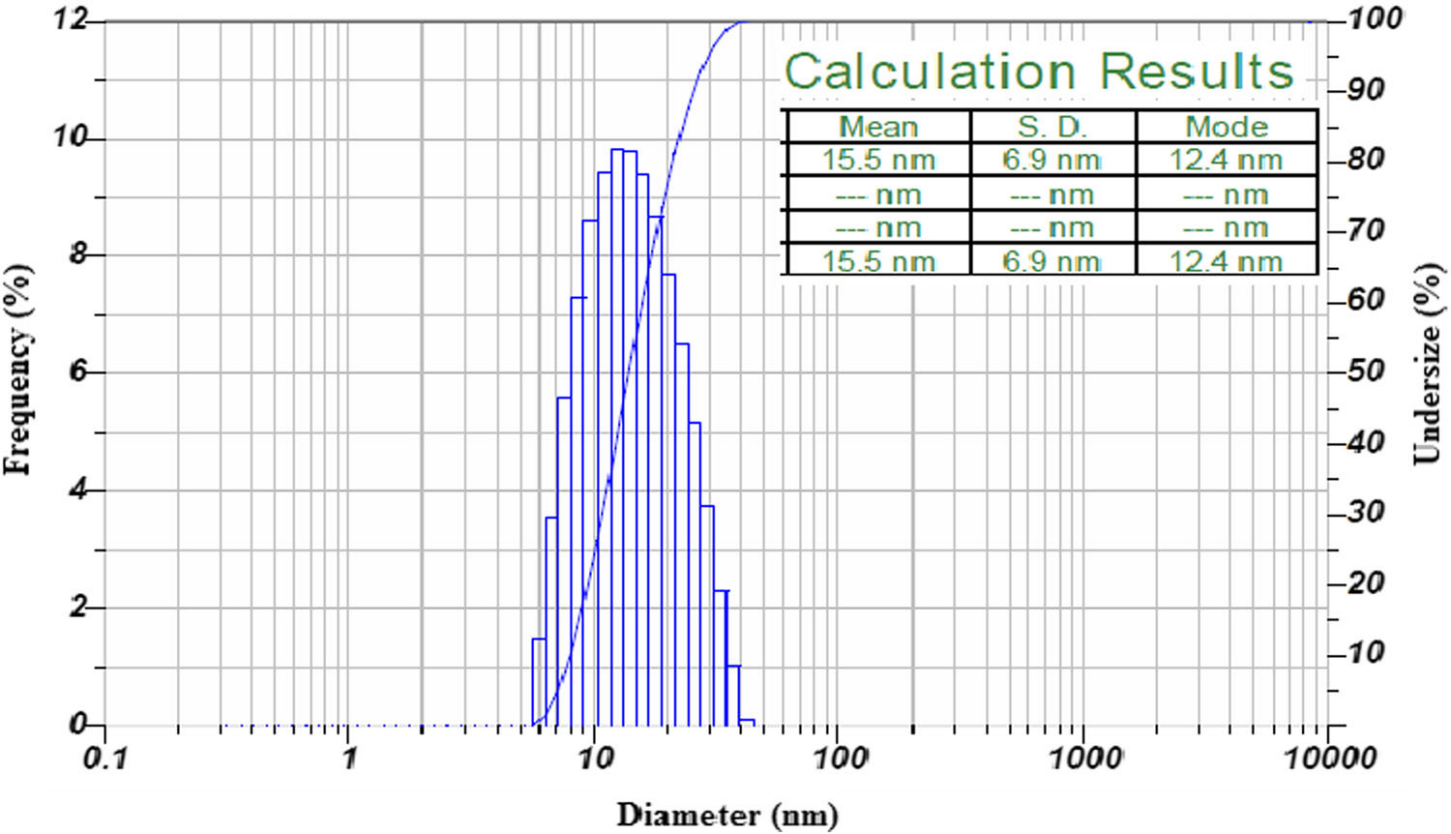
Particle size analysis of biosynthesized AgNPs

The particle size of the biosynthesized AgNPs was detected by intensity and laser diffraction. After the analysis of AgNPs, it has been revealed that the particles synthesized with extracellular fungal filtrate are in the range of 5–40 nm in size. The average size (hydrodynamic radius) of AgNPs was found to be 15.5 nm (Fig. 8). The results are similar to that of TEM results indicating that the size of the AgNPs is uniform in nature. The biosynthesized AgNPs are poly-dispersed in nature. The stability was further confirmed by zeta potential of the particles.

### Zeta potential of AgNPs

**Fig. 9:**
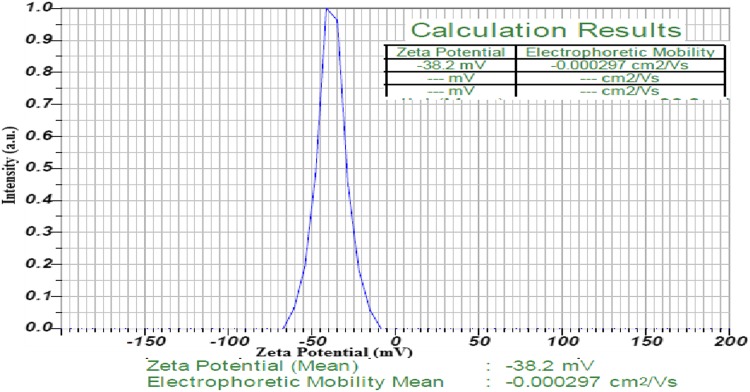
Zeta potential analysis of biosynthesized AgNPs

The electrostatic repulsive force between the nanoparticles depends on the charge which is present on the surface of the nanoparticles. In the present study, the very high negative value of zeta potential confirms the repulsion among the particles and thereby increases the stability of the formulation, and prevents the nanoparticles from agglomeration in the medium, leading to the long-term stability. The zeta potential of the AgNPs of fungal filtrate was found to be −38.2 mV (Fig. 9). Based on the above results, it is concluded that the AgNPs synthesized with *A. versicolor* fungal filtrate were very stable.

### Free radical scavenging activity

Free radical scavenging activity of the AgNPs was checked by DPPH radical scavenging assay. This method is dependent on the reduction of DPPH radical to the non-radical form DPPH–H in the presence of a hydrogen-donating antioxidant. The radical scavenging activity and IC_**50**_ values of all the tested samples were represented in Table 1. The radical scavenging activity was increased with the increasing concentrations of tested samples. The radical scavenging activity for the AgNPs at 100 µg/ml was determined as 60.04 % which is close to 68.52 % obtained for the standard ascorbic acid at the same concentration. However, the radical scavenging activity for the fungal filtrate was found to be 41.19 %. Thus, the biosynthesized AgNPs were proved to be very effective scavengers. The radical scavenging activity was also expressed as IC_**50**_ (inhibitory concentration of the test sample to scavenge 50 % radicals). IC_**50**_ value for the AgNPs is found to be 60.64 µg/ml, while same for the fungal filtrate was found to be 144.84 µg/mL. The lower IC_50_ value means high radical scavenging activity of the sample. Thus, the biosynthesized AgNPs found to be very good antioxidants. Free radical scavenging activity of the AgNPs is mainly due to hydrogen-donating molecules, such as polyphenols, flavonoids, terpenoids, proteins, and other biomolecules, that are present in the colloidal solution of AgNPs or the secondary metabolites, proteins, and other biomolecules that are involved in the synthesis and stabilization of AgNPs. Silver nitrate could not show any free radical scavenging activity in our study. Silver nitrate does not have any hydrogen to participate in the scavenging of free radical DPPH. There were no reports also for the free radical scavenging activity of silver nitrate. Free radical scavenging activity studies of AgNPs biosynthesized in this study are in line with many earlier reports (Ramamurthy et al. 2013). Antioxidants are the compounds which protect cells against the cellular damaging effects of reactive oxygen species (ROS), such as hydroxyl radicals, peroxy radicals, peroxylnitrile, super oxide, and singlet oxygen. The lower concentration of antioxidants or the imbalance between antioxidants and ROS results in oxidative stress which could be linked to inflammation, neurodegenerative disorders, atherosclerosis, aging, and cancer (Ramamurthy et al. 2013). Based on our results, it is revealed that the AgNPs can be used as ingredients in the antioxidant formulations in pharmaceutical or biomedical field. The excellent radical scavenging activity of the AgNPs allowed them to be employed in the numerous applications, such as food preparation ingredients, food storage additives, vegetable oil preparations, and various syrup formulations.

**Table 1:** DPPH radical scavenging activities of AgNPs compared with fungal filtrate and standard ascorbic acid

Tested sample	Radical scavenging activity ± SD (%)				
	25 (µg/mL)	50 (µg/mL)	75 (µg/mL)	100 (µg/mL)	IC_50_
Ascorbic acid	47.08 ± 0.26	55.62 ± 0.25	62.51 ± 0.35	68.52 ± 0.67	33.03
Fungal filtrate	27.05 ± 0.42	31.48 ± 0.34	37.05 ± 0.51	41.19 ± 0.26	144.84
AgNPs	39.28 ± 0.25	47.53 ± 0.19	55.39 ± 0.44	60.04 ± 0.25	60.92

### Antimicrobial activity

**Fig. 10:**
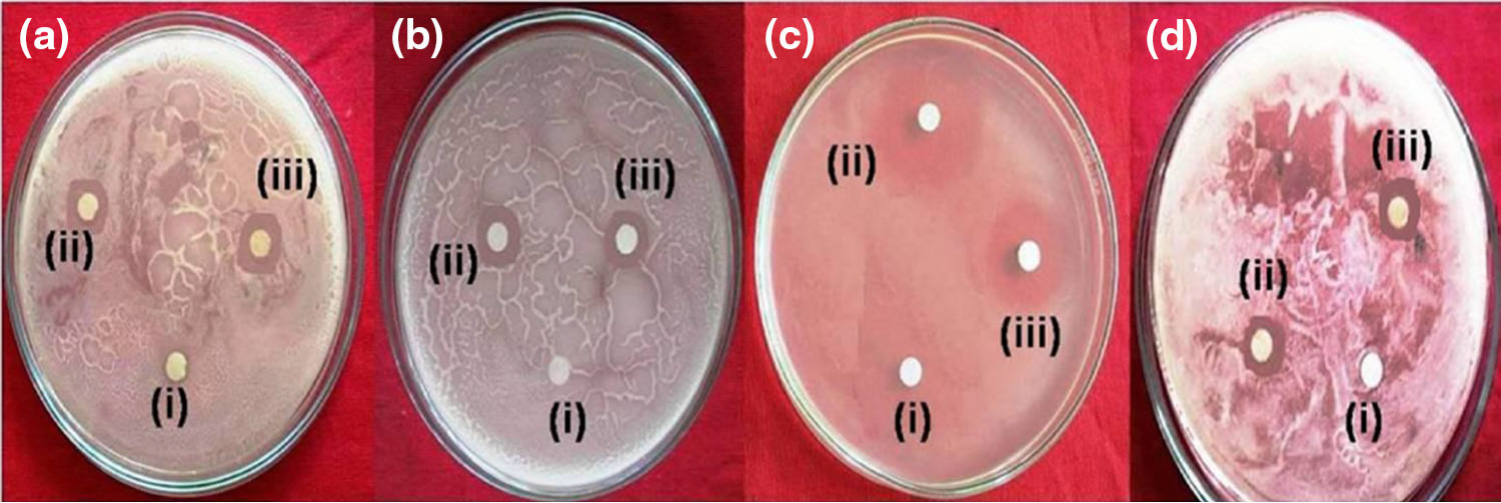
Antibacterial activity of the AgNPs against *S. aureus* (**a**), *S. pneumonia* (**b**), *P. aeruginosa* and **d** *K. Pneumonia*. No inhibition zones formed by fungal filtrate (*i*) Very well inhibition zones were observed by Streptomycin (*ii*) and by AgNPs (*iii*)

After incubation for 24 h, growth inhibition (ZOI) was observed around discs impregnated with AgNPs and streptomycin. Figure 10 shows the strong antibacterial activity of AgNPs against *Staphylococcus aureus, Streptococcus pneumonia* (gram negative), *Pseudomonas aeruginosa*, and *Klebsiella pneumonia* (gram negative); maximum ZOI was observed against *Pseudomonas aeruginosa* (18.5 mm) followed by *Klebsiella pneumonia* (13.6 mm), *Staphylococcus aureus* (12.1 mm), and *Streptococcus pneumonia* (11.8 mm). The results revealed that AgNPs showed strong inhibition against gram-negative bacteria compared to gram-positive bacteria. Not only antibacterial activity, AgNPs also showed a very good antifungal activity against most pathogenic *Candida albicans* and *Candida nonalbicans* and formed the ZOI values of 12.2 and 13.6 mm, respectively. The results revealed that the growth inhibitory activity of AgNPs against fungal pathogens can be increased further by increasing the concentration of AgNPs as these are clean, non-toxic, and biocompatible, but the drugs will be toxic and non-biocompatible at higher concentrations. The results of the antifungal activity were consisted with many earlier reports (Kim et al. 2009; Rafie et al. 2012). The antimicrobial activity of AgNPs is probably the sum of distinct mechanisms. AgNPs react with thiol (-SH) groups of vital enzymes/proteins and inactivates them, in turn stop the progress of cellular metabolisms which leads to cell death (Morones et al. 2005; Netala et al. 2015b). AgNPs form strong interaction with proteins of the proton pump and phospholipid portion of the bacterial membrane resulted in the dissipation of membrane proton gradient causing disruption of bacterial membranes (Kotakadi et al. 2014). AgNPs form strong interaction with respiratory chain enzymes which causes uncoupling of electron transport chain (ETC) and oxidative phosphorylation (OP) as a result cell death occurs (Morones et al. 2005; Feng et al. 2000). Proteomic analysis revealed that the exposure of bacteria to nanosilver resulted in alterations in the expression of envelope and HSPs, as a result AgNPs can penetrate and can disrupt the membranes and leads to leakage of protons, potassium ions and reducing sugars and eventually leads to death (Lok et al. 2006). The excellent antimicrobial activity of the AgNPs allows them to be used in ingredients of topical ointments to prevent bacterial and fungal infections against open and burn wounds, AgNPs embedded water purifier equipments, preparation of injection molded plastic products, and employed in coating-based applications, including air ducts and counter tops. The important advantages of AgNPs-based antimicrobial agents are their biocompatibility, health and environmental safety, and their excellent stability.

## Conclusion

An endophytic fungal isolate, ENT7 was isolated from the leaves of *Centella asiatica* and was identified as *Aspergillus versicolor* based on the molecular characterization of 18 s rRNA gene. A simple and environmental benign approach for the biosynthesis of AgNPs using extracellular filtrate of *Aspergillus Versicolor* ENT7 as reducing agent has been reported. The synthesized AgNPs were well dispersed with the average size of 15.5 nm, spherical in shape, high crystalline in nature, and highly stable with zeta potential of -38.2 mV. The biosynthesized AgNPs exhibited a very good antioxidant and antimicrobial activity and clearly indicated their pharmaceutical and biomedical importance.
